# Vaping induced pneumonitis: a small community hospital’s case series and analysis

**DOI:** 10.1186/s12890-020-1158-2

**Published:** 2020-05-04

**Authors:** Andrew L. Silverman, Haseeb Siddique, Vikas Kumar, Thuy-Hong Le, Joseph Ng

**Affiliations:** Donald and Barbara Zucker School of Medicine at Hofstra/Northwell, Mather Hospital/Northwell Health, 75 N Country Road, Port Jefferson, NY 11777 USA

**Keywords:** E-cigarettes, Pneumonitis, Vaping, Cannabinoids (THC)

## Abstract

**Background:**

Electronic cigarettes/e-cigarettes (ECs), or vaping, is currently the most popular form of smoking amongst youth in the United States. ECs are battery-powered devices that vaporize a liquid that comes in small cartridges, or pods, that contain various chemicals, nicotine, and an array of flavors that can be modified to include cannabinoids (THC). With increasing popularity, however, there is an epidemic of pulmonary and gastrointestinal illnesses associated with vaping in the continental U.S.A.

**Methods:**

We analyzed medical charts of three patients who were active users of ECs and presented with pneumonitis to our community medical center between January and August 2019.

**Results:**

We report three cases of vaping pneumonitis in young adults, ages 18 to 21, who presented with similar symptoms, profiles, imaging studies, and disease progression. The average length of stay was approximately one week, and all patients had an extensive work-up in addition to a relapsing and remitting course of their condition.

**Conclusions:**

Early recognition and diagnosis of vaping pneumonitis are essential in the treatment of the ongoing epidemic. Extensive unnecessary work up may lead to increased healthcare costs. Our case series echoes the concerns of the CDC such that ECs should be avoided, and those with any pulmonary or gastrointestinal symptoms should seek medical attention promptly.

## Background

The prevalence of ECs, or vaping, is increasing worldwide. Data shows increased use of e-cigarettes between 2011 to 2018 of 1.5 to 20.8% among high school students, an estimated increase from approximately 220,000 to 3.05 million high school students. Between 2017 and 2018, e-cigarette use was found to have increased 78% [1]. ECs are marketed as the “safe alternative” to smoking due to the belief that vaping could be a “harm reduction” alternative to smoking to the same degree as other Food and Drug Administration (FDA)-approved nicotine replacement therapies [2]. Vaping companies promote consumer attestations that ECs helped them quit smoking and improved their overall health [3]. These proclamations are controversial as there is sparse literature to show that EC users were more likely to quit smoking than regular cigarette users [4, 5]. There is a concern that ECs could increase worldwide nicotine dependence, especially among young adults who are enticed by the many flavors ECs offer [6]. The medical community is apprehensive of vaping and advises caution since limited scientific evidence is available to show their efficacy and safety [6]. We present three cases of pneumonitis seen at our small community hospital that further supports the harmful effects of ECs.

## Methods

Our 248-bed hospital is located in Port Jefferson, NY, and has about 40,000 ED visits and 12,000 admissions per year. We retrospectively analyzed medical charts of three patients who were active users of ECs and presented with pneumonitis to our community medical center between January and August 2019.

### Cases

#### Case 1

**Fig. 1:**
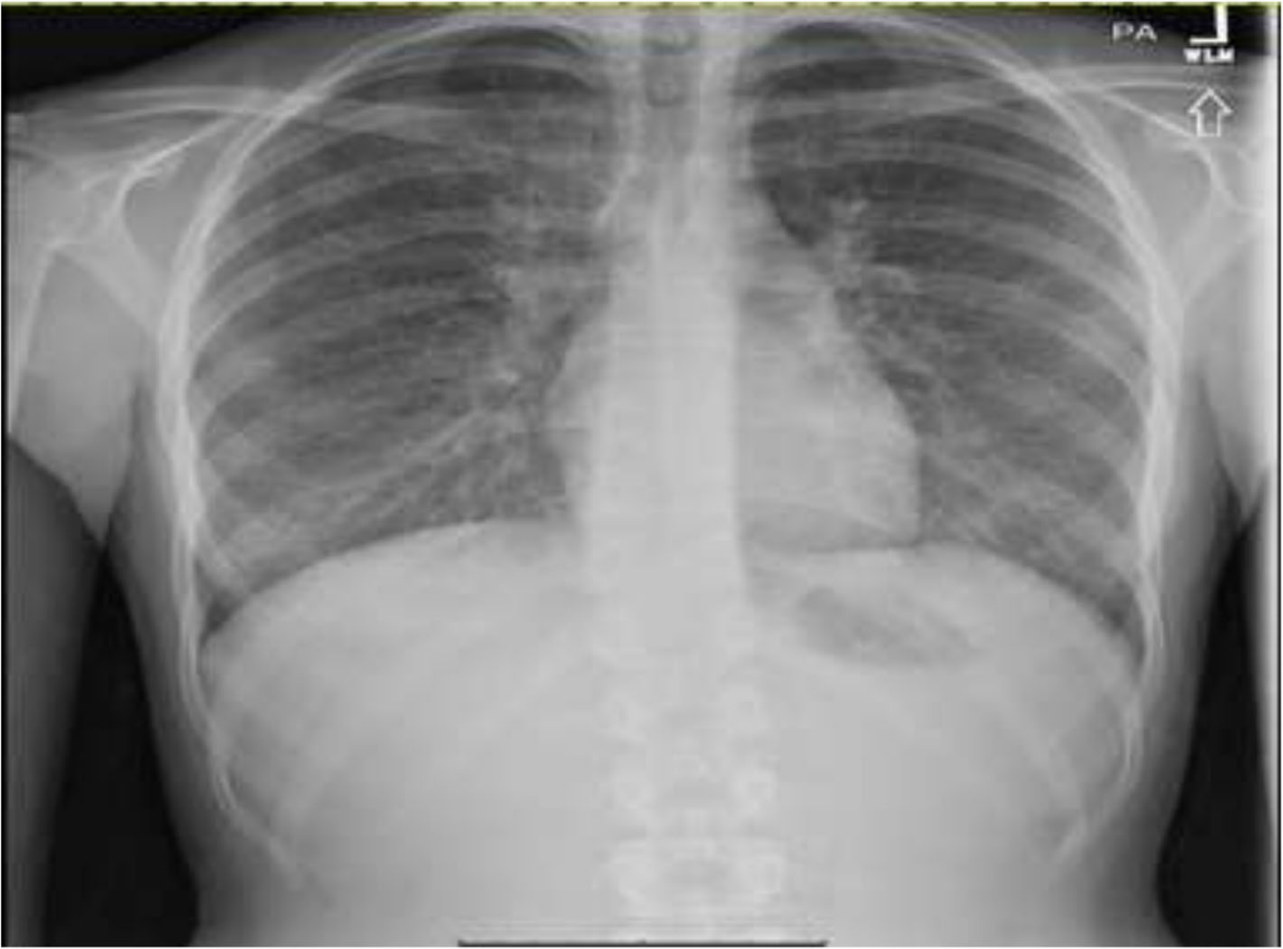
CXR: Coarsening of interstitial lung markings with reticulonodular opacities scattered throughout the lung fields bilaterally

18-year-old previously healthy female with daily THC vaping (1 pod per day for 1 year duration of vaping with THC) use presented to the emergency department (ED) with gastrointestinal symptoms, fatigue, and dyspnea for four days. The patient first had 4–5 episodes of watery diarrhea with diffuse abdominal pain, then nausea with dry heaves, which progressed to non-bloody, non-bilious (NBNB) vomiting. She was given intravenous (IV) fluids and discharged with the diagnosis of a viral illness. However, she returned to the ED the next day with continued abdominal pain and new symptoms of fevers, chills, decreased appetite, and dysuria. She was tachycardic with a heart rate (HR) of 112 beats per minute (BMP), respiratory rate (RR) of 22 breaths per minute (BRPM), oxygen saturation (SpO2) of 95% on 2 L of nasal cannula, and a max temperature of 103 °F. She had leukocytosis with a white blood cell count (WBC) of 17,400 cells per microliter, with 92.8% neutrophils, 3.6% lymphocytes, 2.0% monocytes, and 0.2% eosinophils. Procalcitonin was 0.25 ng/ml, LDH not reported, lactate and lipase were negative. Urine analysis showed positive only for moderate blood and a urine toxicology positive only for cannabinoids. Recent HIV and STD testing were negative. Chest X-Ray (CXR) showed bilateral reticulonodular opacities (Fig. 1).

The patient was admitted and started on broad-spectrum antibiotics to treat potential pneumonia (PNA). She tested negative for both influenza A and influenza B. Tests for C. Diff, HIV, p-ANCA, c-ANCA, and ANA were all negative. Mycoplasma pneumoniae, Chlamydia psittaci, Chlamydia pneumoniae, and Legionella were also negative. Treatment was supportive with oxygen therapy, steroids, antibiotics, antiemetics, and pain medication. The patient was on steroids for a total of 6 days while hospitalized and then discharged on a prednisone taper. The patient reported an overall improvement in her symptoms upon discharge on day 6.

#### Case 2

18-year-old male with a remote history of childhood asthma and 2-years of daily vaping marijuana who presented with dyspnea, fever, nausea, vomiting, and abdominal pain for four days. The patient’s symptoms began with substernal, pleuritic chest pain. He then developed chills and vomiting (NBNB) of 7–8 episodes. The morning before presenting to the hospital, the patient went to an urgent care clinic for his worsening symptoms, but was then sent to the ED. His max temperature was 100 °F, blood pressure (BP) was 99/72 mmHg, HR of 116 BPM, respiratory rate (RR) of 19 breaths per minute (BRPM), oxygen saturation (SpO2) of 94% on ambient room air. Laboratory findings were notable for leukocytosis with a white blood cell count (WBC) of 24,900 cells per microliter, with 95.3% neutrophils, 1.6% lymphocytes, 2.1% monocytes, and 0.0% eosinophils. Procalcitonin was 0.45 ng/ml, LDH 400 ng/ml, D-Dimer 406.91 ng/mL, lactate negative, and lipase negative. CT chest notable for bilateral ground-glass opacities throughout both lungs and prominent lymph nodes.

**Fig. 2:**
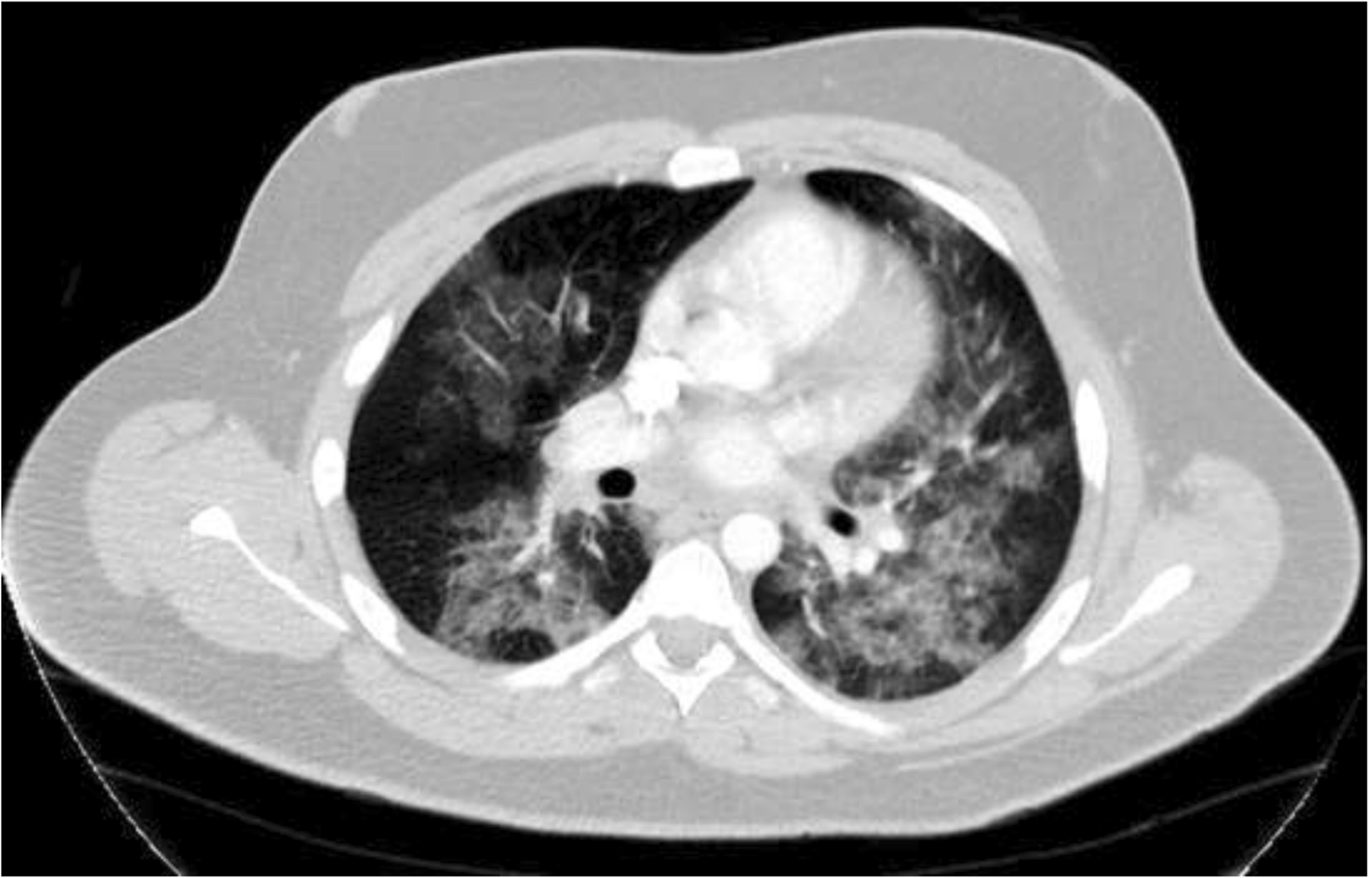
Axial CT Chest Scan with IV contrast: Diffuse bilateral ground-glass opacities throughout both lungs, demonstrating predominantly a perihilar distribution, with areas of mild bronchial wall thickening. Several mildly prominent hilar lymph nodes, the largest measuring 1.2 cm in the short axis on the right

The patient was admitted and started on 2 L of normal saline, azithromycin and ceftriaxone for community-acquired PNA, Zofran as antiemetic, and morphine 4 mg IV for pain while in the ED. Urine toxicology done after receiving IV morphine for pain was positive for cannabinoids and opiates. During his hospital course, extensive infectious gastrointestinal and pulmonary workup were conducted, and all results were negative, including but not limited to, blood cultures, stool sample, HIV, Influenza A and B, Norovirus, and respiratory viral panel. Troponins and D-dimer were negative. Autoimmune workup, including ANA, p-ANCA, c-ANCA, and celiac testing, were negative. Esophagogastroduodenoscopy showed a hiatal hernia and possible Barrett’s esophagus/eosinophilic esophagitis. Abdominal ultrasound and a transesophageal echocardiogram were negative. Radiographic and CT imaging were inconclusive for pneumonia vs. pneumonitis (Fig. 2). Treatment was supportive with oxygen therapy, steroids, antibiotics, antiemetics, and pain medication. Steroids were given for the entire nine-day duration of hospitalization and he was discharged on a steroid taper. Clinically, the patient reported an overall improvement in his symptoms upon discharge on day nine.

#### Case 3

21-year-old male with a past medical history of four months of daily EC vaping, 2 pack-years cigarette smoking history, anxiety, bipolar disorder, and asthma presented for worsening dyspnea. Three days before admission, he presented to another hospital ED, where he was prescribed Augmentin. The patient had a continuation of symptoms and went to urgent care where he had a CXR, which showed possible pneumonia and was instructed to proceed to the hospital.

**Fig. 3:**
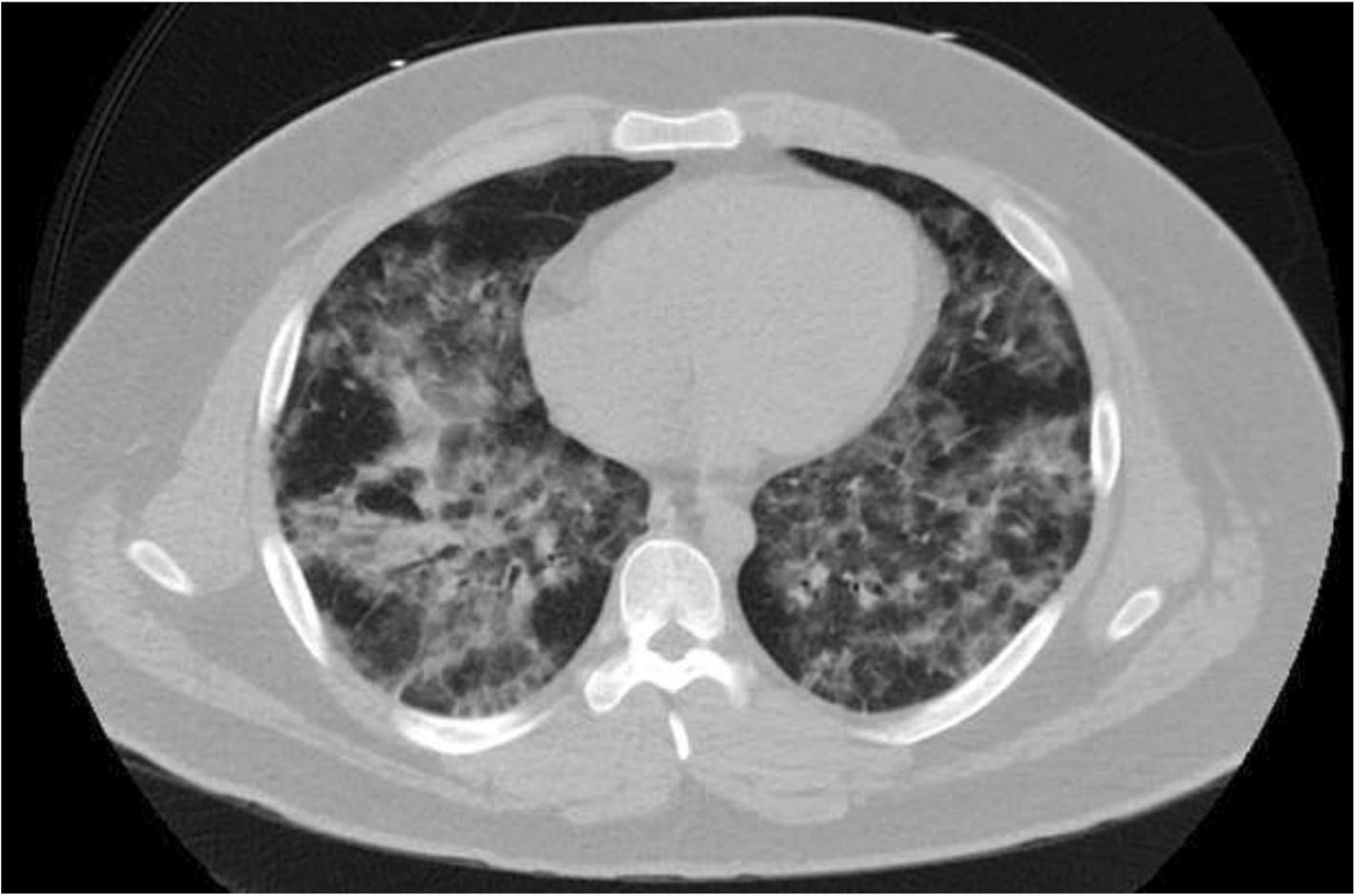
Axial CT Chest without IV Contrast: Diffuse interlobular septal thickening and ground-glass opacities involving the bilateral lung fields

Vitals on presentation as follows: temperature was 99 °F; BP was 135/91 mmHg; HR of 120 BPM; RR of 20 BRPM; SpO2 88% on ambient room air. Laboratory findings were notable for leukocytosis with a white blood cell count (WBC) of 16,500 cells per microliter, with 91.2% neutrophils, 4.5% lymphocytes, 2.8% monocytes, and 0.8% eosinophils. Procalcitonin was negative, LDH was not recorded, and lactate was negative. The patient was admitted for acute hypoxic respiratory failure. He was admitted to the ICU and placed on a high flow nasal cannula for potential respiratory compromise. Duonebs, 2 L of normal saline bolus, and antiemetics were initiated. CXR showed evidence of potential small airspace consolidation in the right lower lobe (Fig. 3). Broad-spectrum IV antibiotics for pneumonia coverage and steroids were started. Blood, urine, and sputum samples were all negative. Toxicology screening was positive for cannabinoid use and benzodiazepine use, which were expected from vaping and Xanax prescriptions. While hospitalized, the psychiatry team adjusted the patient’s psychiatric medications. Treatment was supportive with oxygen therapy, steroids, antibiotics, antiemetics, and pain medication. Steroids were given during the entire eight-day hospitalization and he was discharged on a steroid taper. The patient clinically improved on day 8 and was discharged home with oxygen.

## Results

There were 3 cases of patients aged 18–21. All three patients presented with bilateral opacities on imaging and respiratory symptoms. The average length of stay was one week and all patients had extensive medical work up that is not part of routine lab work, including imaging and bloodwork, to rule out infectious etiology such as pneumonia, workup to rule out autoimmune diseases such as Celiac Disease, urine toxicology, arterial blood gas and antibody testing (Table 1). Patients’ workups included numerous consults, including Infectious Disease, Pulmonology, Gastroenterology and Psychiatry. All three patients endorsed vaping, but two of the three patients (66.67%) endorsed using THC vaping products. With aggressive supportive care, none of the patient’s required mechanical ventilation. All patients improved with steroids and supportive treatment after a relapsing and remitting course.

**Table 1:** Demographic information and lab test results for patient 1, patient 2, patient 3

	Patient 1	Patient 2	Patient 3
Age/Gender	18/F	18/M	21/M
History of vaping	+	+	+
Fever	+	+	–
Rapid respiratory viral panel*	N/A	–	–
Influenza A/B	–	–	N/A
HIV	–	–	N/A
Mycoplasma pneumoniae	–	–	N/A
Chlamydia psittaci, chlamydia pneumoniae	–	–	N/A
*Streptococcus pneumoniae*	N/A	–	–
Legionella	–	–	–
Shigella	N/A	–	N/A
Giardia	N/A	–	N/A
Cyclospora, ova/parasites	N/A	–	N/A
*Clostridium difficile*	–	–	N/A
Norovirus	N/A	–	N/A
IgE	WNL	+	WNL
ANA	–	–	N/A
p-ANCA/c-ANCA	–	–	–
ABG showing low oxygen saturation	N/A	N/A	+
Imaging showing bilateral opacities	+	+	+
Urine toxicology report	Positive for cannabinoids	Positive for cannabinoids and opiates	Positive for cannabinoids and benzodiazepines
Length of stay inpatient	6 days	9 days	8 days

*WNL* indicates within normal limits

*N/A* indicates not applicable (not tested)

*Rapid respiratory viral panel tested for: adenovirus; coronavirus (HKU1, NL63, 229E, OC43); human metapneumovirus (hMPV); human enterovirus/rhinovirus (Entero/RV); influenza A; influenza A/H1; influenza A/H3; influenza A/H1–2009; influenza B; parainfluenza viruses 1, 2, 3, 4; respiratory syncytial virus; *Mycoplasma pneumoniae*; and *Chlamydophilia pneumoniae*

## Discussion

One thousand eighty cases of vaping induced pneumonitis have been reported to the CDC as of October 1st, 2019. These cases come from 48 different state health departments and 1 U.S. territory. All patients had a history of e-cigarette use. The course of the disease process typically begins with pulmonary symptoms of nonproductive cough, pleuritic chest pain, and/or shortness of breath lasting over several days to weeks before the patient is hospitalized. All patients described in these reports had abnormal imaging that included infiltrates on chest radiograph and ground-glass opacities on chest CT scan. Gastrointestinal findings include nausea, vomiting, abdominal pain, and diarrhea have also been seen in these patients. Many patients, due to the nonspecific symptoms, received an initial diagnosis of infection and were treated with empiric antibiotics, which did not lead to improvement. One of the most severe symptoms that led to hospitalization was hypoxemia, which in some cases progressed to acute or subacute respiratory failure [2]. Patients in these cases needed multiple supplemental therapies, including supportive oxygen, endotracheal intubation, or even mechanical ventilation. The therapy that showed the most improvement in these patients was corticosteroids.

This case series, at a small community hospital, describes a possible correlation between vaping and pulmonary pneumonitis in three young adult patients. Each patient had a history of vaping and had similar imaging findings. The average length of stay was approximately one week, and all patients had a relapsing and remitting course of their condition. Extensive workup with multiple negative results led to increased healthcare costs that are preventable with a better understanding of the presentation of vaping induced pneumonitis.

It should be noted that the imaging findings in these patients are similar to those seen in patients with acute eosinophilic pneumonia due to cigarette smoking. Both vaping induced pneumonitis and acute eosinophilic pneumonia have imaging that resemble bacterial pneumonia. Both diseases cause a wide range of symptoms, including fever, cough and shortness of breath. They can each progress to necessitating ICU admissions. However, a main difference between the two conditions revolves around the research that has been conducted to this point. The main mechanism of acute eosinophilic pneumonia has been elucidated to be due to pro-inflammatory cytokines such as IL-5, IL-6, IL-7, and tumor necrosis factor. This combination of pro-inflammatory cytokines is thought to be the inciting event in this disease process, which leads to the eosinophil-rich exudate within the alveoli [7, 8]. The main mechanism of action of vaping induced pneumonitis, however, has not been elucidated to this point. Further research may focus on elucidating this mechanism so as to create better treatments for this condition.

On September 6, 2019, the CDC issued warnings to cease the use of all vaping products due to an outbreak in severe lung-related illnesses [9]. According to FDA reports, e-cigarette cartridges and solutions contain a mixture of contaminants that can be harmful to humans, including nitrosamines and diethylene glycol [10]. THC, the principal psychoactive component of cannabis, is noted by the CDC to be a product that can be delivered via e-cigarettes [1]. Of note, cannabis was a consistent finding among the patients in this case series. No e-cigarette product, substance, or additive was consistently identified among the cases that have been reported [9, 11]. Therefore, it can be hypothesized that THC containing e-cigarettes may be a principal cause in the pathophysiology of vaping induced pneumonitis, even if no specific substance has been identified as the principal cause thus far.

## Conclusions

Recently in the New England Journal Of Medicine, a study of 53 cases were reported with vaping induced pulmonary injury, similar to the cases presented at our community hospital [12]. Upon reviewing this retrospective case series, our findings further support the CDC’s recommendations to cease vaping until the etiology of lung injury is determined. No single substance or chemical is linked to the cause as of yet. More cases are being discovered around the world.

## Data Availability

All authors had access to data and material and vouch for its complete accuracy. Literature review can be accessed through New England Journal of Medicine coinciding with our findings in our Case Series. Data and Materials can be accessed through the Medical Records at Northwell Health Mather Hospital in the medical chart. Consent forms have been signed and are available as hard copies. All images are available through PACS Imaging system storage at Northwell Health, Mather Hospital.

## Abbreviations

EC: Electronic Cigarettes
THC: Tetrahydrocannabinol
FDA: Food and Drug Administration
NBNB: Non-Bloody, Non-Bilious
IV: Intravenous
HR: Heart Rate
BMP: Beats per minute
WBC: White blood cell count
CXR: Chest X-Ray
C.Diff: *Clostridium difficile*
HIV: Human Immunodeficiency Virus
p-ANCA: Perinuclear antineutrophil cytoplasmic antibodies
c-ANCA: Cytoplasmic antineutrophil cytoplasmic antibodies
ANA: Antinuclear Antibody
RR: Respiratory Rate
